# Acute severe ulcerative colitis: management advice for internal medicine and emergency physicians

**DOI:** 10.1007/s11739-021-02704-0

**Published:** 2021-03-22

**Authors:** Konstantina Rosiou, Christian Philipp Selinger

**Affiliations:** 1 Leeds Gastroenterology Institute, Leeds Teaching Hospitals NHS Trust, St James University Hospital, Bexley Wing, Leeds, LS9 7TF UK; 2 University of Leeds, Leeds, UK

**Keywords:** Ulcerative colitis, Inflammatory bowel disease

## Abstract

Acute severe ulcerative colitis is a medical emergency that warrants in-patient management. This is best served within a multidisciplinary team setting in specialised centres or with expert consultation. Intravenous corticosteroids remain the cornerstone in the management of ASUC and should be initiated promptly, along with general management measures and close monitoring of patients. Unfortunately, one-third of patients will fail to respond to steroids. Response to intravenous corticosteroid therapy needs to be assessed on the third day and rescue therapies, including cyclosporine and infliximab, should be offered to patients not responding. Choice of rescue therapy depends on experience, drug availability and factors associated with each individual patient, such as comorbidities, previous medications or contra-indications to therapy. Patients who have not responded within 7 days to rescue therapy must be considered for surgery. Surgery is a treatment option in ASUC and should not be delayed in cases of failure of medical therapy, because such delays increase surgical morbidity and mortality. This review summarises the current management of acute severe ulcerative colitis and discusses potential future developments.

## Introduction

Ulcerative colitis (UC) is a chronic inflammatory condition that affects the mucosal surface of the colon, starting from the rectum and extending more proximally in a continuous fashion [1]. Its incidence is rising worldwide [2] and its precise aetiology is unknown and believed to be multifactorial, involving genetic predisposition, epithelial barrier defects, dysregulated immune responses to luminal pathogens as well as environmental factors [3]. Ulcerative colitis is characterised by a relapsing and remitting course [3] and although the majority of patients tend to have a mild to moderate disease course, approximately 15–25% of patients with UC will experience at least one episode of severe flare of their disease and 10–20% will present with acute severe ulcerative colitis (ASUC) at diagnosis [4, 5]. Despite improvements in management, ASUC should still be considered a medical emergency. ASUC is associated with a 20% risk for colectomy on first admission and this risk rises to 40% after two admissions [4]. The risk of colectomy in ASUC has also been related to the biological severity at admission based on the fulfilment of the Truelove and Witts criteria, the extent of the disease either at diagnosis or follow-up, male gender and the presence of extra-intestinal manifestations of inflammatory bowel disease (IBD) [4]. Moreover, there remains a 1% mortality associated with severe flares of UC [6, 7]. Medical management of ASUC remains a significant challenge for clinicians. The aim of this review is to underline the key principles in the diagnosis and management of ASUC, highlighting at the same time useful advice for internal medicine and emergency physicians involved in the management of patients with ASUC.

## Definition

The diagnosis of acute severe ulcerative colitis is based on the Truelove and Witts criteria as described at their original article in 1955 [8]. Every patient known to have UC presenting with bloody diarrhoea ≥ 6/day and any sign of systemic toxicity, i.e. at least one of: pulse > 90/min, temperature > 37.8 °C, haemoglobin < 105 g/L, erythrocyte sedimentation rate (ESR) > 30 mm/h should be admitted to hospital for assessment and intensive treatment [9, 10]. C-reactive protein (CRP) is measured more often than ESR and does not show a non-specific increase with age [11] and for this reason, a CRP of > 30 mg/L has been incorporated in the modified Truelove and Witts criteria endorsed by both the European Crohn’s and Colitis Organisation (ECCO) and the British Society of Gastroenterology (BSG) [9, 10]. (Table 1).

**Table 1:** ASUC definition [8, 10]

Bloody diarrhoea > 6/day AND at least one of:
Heart rate > 90 bpm
Temperature > 37.8 °C
Haemoglobin < 105 g/L
ESR > 30 mm/h
CRP > 30 mg/L

## Initial assessment

All patients admitted with ASUC should have initial investigations aiming to assess disease severity, exclude infections and relative contra-indications of rescue therapy and predict outcomes [12]. Baseline investigations are presented in Table 2 and include: full blood count (FBC), CRP, urea and electrolytes (U&E), liver function tests (LFT’s), magnesium, stool cultures and *Clostridium difficile* (*C. difficile*) assay, radiological imaging (abdominal X-ray or computed tomography/CT) and flexible sigmoidoscopy.

**Table 2:** Baseline investigations

Full blood count, urea and electrolytes, liver function tests, CRP, magnesium
Stool cultures including *Clostridium difficile* assay
Plain abdominal X-ray
Sigmoidoscopy including CMV screen
Screening tests for 2nd-line therapy (if not already performed):
HBV, HCV, HIV, VZV, screening for tuberculosis (chest X-ray, interferon-gamma release assay), lipid profile, TPMT

Stool cultures and microscopy should be performed as early as possible upon admission to exclude infectious diarrhoea and test for *C. difficile* toxin. *C. difficile* infection is associated with increased morbidity and mortality in UC patients, as well as increased risk for surgery and healthcare related costs [13–15]. At the moment, there is no current recommendation as to whether one sample or serial testing for *C. difficile* should be performed and there are studies supporting both sides [16, 17]; however, if *C. difficile* is detected, oral vancomycin 500 mg/6 h for 10 days should be given [10].

Radiological imaging plays an important role in the management of patients with ASUC. Plain abdominal X-ray should be requested on admission and serial X-rays are used to monitor disease progression or complications. Toxic megacolon, defined by a colonic diameter of > 5.5 cm in the presence of systemic toxicity, represents a life-threatening complication of ASUC and in the majority of cases requires immediate surgery. A plain abdominal X-ray can also provide an estimate of the extent of the disease, as the proximal extent of disease correlates with the distal distribution of faecal residue. Moreover, there are predictors of poor prognosis, such as a small bowel dilation > 3 cm, the presence of ≥ 3 gas-filled loops of small bowel or the visualization of small circular opacities, which represent residual isolated mucosa surrounding an ulceration, that can be assessed with an abdominal radiograph [18]. Routine CT scans have minor impact on the decision to perform colectomy and are not recommended [19]. CT plays a vital role in the detection of complications, especially perforation, while Magnetic-Resonance Imaging (MRI) and ultrasound may be utilised to determine the extent of disease [10].

Early unprepped flexible sigmoidoscopy can aid to confirm diagnosis, assess disease severity and obtain histology, including ruling out Cytomegalovirus (CMV) infection [9, 10]. A complete colonoscopy is not advised as it carries an increased risk of perforation in patients with ASUC and does not offer more clinical information compared to a sigmoidoscopy [9].

CMV infection can be diagnosed by the presence of CMV inclusion bodies on haematoxylin and eosin (H&E) biopsy staining, by immunohistochemistry and/or tissue Polymerase chain reaction (PCR) [10]. The prevalence of CMV infection during ASUC ranges among different studies; however, it can be over 30% among steroid-refractory patients [20]. Empirical antiviral therapy should be initiated in patients with moderate-to-severe colitis and a high suspicion of CMV infection (steroid-resistant, severe immunosuppression, etc.) [21]. Those with proven colonic CMV reactivation require antiviral therapy in all cases [21]. Current guidelines recommend treatment with intravenous ganciclovir 5 mg/kg twice daily for 3–5 days, which should be then switched to oral valganciclovir 900 mg twice daily for 2–3 weeks. In case of systemic CMV reactivation causing either meningo-encephalitis, pneumonitis, hepatitis, oesophagitis or colitis, discontinuation of all immunosuppressive medication is advised along with antiviral treatment [22].

Flexible sigmoidoscopy can be utilised to rate the severity of mucosal inflammation. The most commonly used scoring systems are the *ulcerative colitis endoscopic index of severity *(*UCEIS*) and the *endoscopic Mayo score*. The use of endoscopic scoring systems can aid to uniformity at initial assessment as well as follow-up [23, 24]. Moreover, endoscopic scoring systems can be useful in predicting disease outcome. For example, a UCEIS of 7 or 8 is a strong predictor of the need of rescue therapy, colectomy or readmission and this can be useful in early decision-making [25].

Screening tests for second-line therapy are required for all patients unless these tests were already performed as an outpatient prior to presentation with ASUC and include lipid profile, hepatitis B and C virus, human immunodeficiency virus (HIV), varicella zoster virus (VZV) (if no history of chicken pox, shingles or varicella vaccination), screening for tuberculosis with clinical risk stratification, chest X-ray and interferon-gamma release assay [10]. Thiopurine methyltransferase (TMPT) should also be measured in view of possible use of thiopurines as maintenance therapy, as it can be useful in predicting patients at risk of developing significant drug toxicity [12].

## General management (Table 3)

**Table 3:** ASUC checklist

✓	Admit all patients with ASUC to a specialised gastrointestinal ward for multidisciplinary care OR seek specialist input at early stage
✓	Exclude other causes of symptoms (including infective, ischaemic or drug-induced colitis)
✓	Assess patients at least once daily (physical examination, haemodynamic status, stool charts, blood tests ± abdominal X-Ray)
✓	Correct and prevent dehydration and electrolyte imbalances
✓	Avoid NSAIDs, anticholinergic, anti-diarrhoeal, opioid drugs
✓	Assess and optimise nutritional status—encourage enteral nutrition
✓	Initiate prophylaxis for VTE
✓	Avoid routine use of antibiotics
✓	Consider topical therapy if tolerated
✓	Maintain a haemoglobin above 8–10 g/dL, prefer intravenous iron supplementation

Patients admitted with ASUC are best managed in the setting of a multidisciplinary team including a gastroenterologist, colorectal surgeon, gastroenterology nurse, dietician, pharmacist and stomal therapist on a specialised gastrointestinal ward. If such care is unavailable, discussion with a specialist centre to establish the management plan at an early stage should be sought [9, 12]. Access to emergency surgery is vital for all patients with ASUC to manage potential complications and for those failing 1st- and 2nd-line medical therapies.

Other causes of symptoms should always be considered and excluded, including infective, ischaemic, or drug-induced colitis [26]. Patients should be assessed at least once daily including physical examination, assessment of haemodynamic status, stool charts, blood tests and abdominal radiographs when required [12, 26]. Intravenous fluids and electrolytes should be replaced as required to correct and prevent dehydration and electrolyte imbalance. Hypokalaemia and hypomagnesaemia should be corrected because they can promote toxic dilatation [9, 27].

Non-steroidal anti-inflammatory drugs (NSAIDs) have been associated with disease exacerbation and should therefore be avoided [9, 27, 28]. Moreover, anticholinergic, anti-diarrhoeal and opioid drugs should be withdrawn because they can precipitate colonic dilatation [9, 27, 29].

The nutritional status of the patient should be assessed preferably by a trained dietician [12] and nutritional support should be instituted for malnourished patients [9]. There is no proven role for routine parenteral nutrition and enteral nutrition is considered more appropriate and associated with less complications [9, 30]. Total bowel rest does not alter outcomes in patients with ASUC and is therefore not recommended [9, 31].

Patients with IBD have a threefold higher risk of venous thromboembolism (VTE) compared to patients without IBD and this risk increases even more during flares of the disease and hospitalisation with need of steroids [32–35]. For this reason, VTE prophylaxis both pharmacological with subcutaneous low molecular weight heparin and mechanical with graduated compression stockings is advised unless contraindicated [9, 12, 36]. The presence of rectal bleeding is not a contraindication to subcutaneous low molecular weight heparin.

Routine use of antibiotics in ASUC is not recommended. Controlled trials of oral or intravenous metronidazole, tobramycin, or ciprofloxacin in patients with ASUC did not show any additional benefit compared to conventional therapy [37–39]. For this reason, antibiotics are only recommended when infection is considered (for example, in a first attack of short duration, after recent admission in hospital or after travel to an area where amoebiasis is endemic) or immediately prior to surgery [9, 40].

There is no definite evidence for continuation of 5-aminosalicylic acid (5-ASA) therapy [26]. However, current guidelines recommend topical therapy (either 5-ASA or corticosteroids) if this can be tolerated by the patient and retained [9].

Blood transfusion is indicated if required to maintain a haemoglobin above 8–10 g/dl [9, 41]. If iron supplementation is needed, then intravenous iron should be preferred and is recommended by current ECCO guidelines as it is more effective, shows a faster response and is better tolerated than oral iron [41].

## Treatment

### Corticosteroids

Ever since the pivotal article of Truelove and Witts, intravenous corticosteroids have been the cornerstone in the management of acute severe ulcerative colitis [8]. Use of intravenous corticosteroids in the management of ASUC has led to a decrease in the morbidity and mortality associated with a severe flare of UC [7, 42]. Current guidelines recommend using methylprednisolone 60 mg every 24 h or hydrocortisone 100 mg four times daily [9, 10, 12, 40]. There is no proven effectiveness from higher doses, but lower doses are less effective, and a bolus injection is as effective as continuous infusion [7, 43, 44]. A systematic review including 1991 patients from 32 trials of steroid therapy for ASUC between the years 1974 and 2006, reported on overall response to steroids (including intravenous hydrocortisone, methylprednisolone and betamethasone) of 67% and this outcome did not change between 1974 and 2006 [7]. Treatment with steroids should be given for a defined period and extending therapy beyond 7–10 days carries an increased risk of toxicity without adding any benefit [7, 45]. According to the current guidelines, patients should be assessed after three days of intravenous corticosteroid therapy and those not responding should be considered for salvage medical or surgical therapy [10, 40].

### Predictors of outcomes in ASUC, response to corticosteroid therapy and indicators for rescue therapy

Several markers have been studied as predictors of outcome in ASUC. These can be divided into clinical, biochemical, endoscopic and radiological and several indices and scoring systems have been developed, that can potentially predict disease outcomes and therefore guide patient management. Low albumin levels have been associated with an increased risk for colectomy [46, 47] and a CRP/albumin ratio of 0.85 on day 3 of intravenous steroid therapy was found to predict the need for colectomy with a sensitivity of 70% and specificity of 76% in one study [48]. Endoscopic markers can also be used as predictors and severe endoscopic lesions including deep ulcers, extensive loss of mucosal layers, well-like ulcers or large erosions have been associated with non-response to corticosteroids [49] and need for colectomy [50]. As previously mentioned, a UCEIS of ≥ 7 on admission is a predictor of the need of rescue therapy or colectomy [25]. The use of faecal calprotectin as a predictor of failure of corticosteroid therapy has also been investigated. A prospective cohort study by Jain et al. found that all patients with UCEIS > 6 on admission and faecal calprotectin > 1000 μg/g on day 3 failed steroid therapy [51].

Up to one-third of patients will fail to respond to intravenous corticosteroids and it is therefore very important that patients are assessed in a timely manner to identify those that need to be considered for salvage therapy either medical or surgical. The most commonly used criteria which are endorsed in current guidelines are the Oxford criteria [10, 52]. These are defined by > 8 stools per day or three to eight stools per day with a CRP > 45 mg/L on day 3 of intravenous steroid therapy and correspond to 85% rate of colectomy [52]. The Oxford criteria are the simplest to apply in clinical practice. The Edinburg risk score described by Ho et al., assesses the mean stool frequency over the first three days of admission, along with the presence of colonic dilatation (> 5.5 cm) and hypoalbuminaemia (< 30 g/L) on the first day of admission. A score of ≥ 4 can predict failure of corticosteroid therapy with a sensitivity of 85% and specificity of 75% [53]. The Swedish index, also known as the fulminant colitis index, is also calculated on day 3 [stool frequency/day + 0.14 X CRP mg/L] and a score of ≥ 8 has a positive predictive value of 72% for colectomy [54]. (Table 4).

**Table 4:** Predictors of failure of corticosteroid therapy or colectomy

To be assessed at day 3 of intravenous corticosteroids		
Travis et al. [52]	> 8 stools/day or 3–8 stools/day AND CRP > 45 mg/L	85% chance of colectomy
Ho et al. [53]	Mean stool frequency of days 1–3, albumin on admission < 30 g/L, colonic dilatation > 5.5 cm on X-ray	85% chance of treatment failure if score ≥ 4
Lindgren et al. [54]	Stool frequency/day + 0.14 × CRP mg/L	72% chance of colectomy if ≥ 8
Gibson et al. [48]	CRP/albumin ratio > 0.85 plus > 3 stools/day	74% chance of treatment failure

According to current guidelines, all patients with ASUC that are assessed by a suitable scoring system and fail to respond to intravenous corticosteroid therapy by day 3 should be offered rescue therapy [9, 10].

## Rescue therapies in ASUC

### Calcineurin inhibitors

Cyclosporine given intravenously was the first agent to be used with proven efficacy as second-line treatment in patients with steroid-refractory severe UC. The first trial on cyclosporine in ASUC by Lichtiger et al. in 1994, demonstrated efficacy of intravenous dose of 4 mg/kg/day [55]. A randomised controlled trial from Belgium showed that cyclosporine given intravenously at 2 mg/kg/day had similar efficacy to the higher dose of 4 mg/kg/day used initially [56]. Current guidelines recommend cyclosporine 2 mg/kg/day as salvage therapy with a target trough cyclosporine concentration of 150–250 ng/mL. Response to cyclosporine should be assessed between days 4–7 and patients who responded should be switched to an oral dose twice the intravenous dose. This is administered in divided doses twice daily with a target trough level of 100–200 ng/mL [10]. Oral cyclosporine should be continued for several months and used as a bridging therapy for the introduction of a thiopurine, either Azathioprine or Mercaptopurine, which will then be used as maintenance therapy after tapering of cyclosporine [10]. Patients who had been treated with thiopurines as maintenance therapy prior to the episode of ASUC and had inadequate response, should not receive cyclosporine as rescue therapy and alternatives should be considered [12].

Cyclosporine use in ASUC has been limited by the relative common side effects associated with its use. Treatment with cyclosporine has been associated with serious infections (6.3%), anaphylaxis (0.9%), nephrotoxicity (5.4%) and neurotoxicity (seizures 3.6%), as well as a mortality of 1.8% [57]. Cyclosporine should be avoided in patients with hypocholesterolaemia (cholesterol level < 1.15 mg/L) and hypomagnesaemia (serum magnesium < 1.4 mg/L) because some of the neurotoxicity seen with the use of cyclosporine has been associated with these two conditions [57]. Nephrotoxicity as indicated by a rise in serum creatinine is typically mild and may respond to lowering of the dose of cyclosporine [57]. Magnesium, cholesterol and creatinine should be measured at baseline and after 48 h of treatment with cyclosporine [58]. Patients who respond to cyclosporine and start maintenance therapy with thiopurines, should also receive prophylactic antibiotic therapy for *Pneumocystis jirovecii* (trimethoprim–sulfamethoxazole 160/800 mg three times a week) while on triple immunosuppression with oral steroids more than an equivalent to 20 mg of prednisolone [22].

Tacrolimus is an alternative calcineurin inhibitor which can be administered orally and has a similar mechanism of action to cyclosporine. There have been only a few studies on the use of tacrolimus in ASUC; however, a systematic review and meta-analysis by Komaki et al. demonstrated the efficacy of tacrolimus, showing that clinical response at two weeks of therapy was higher with tacrolimus compared to placebo (RR = 4.61, 95% CI 2.09–10.17, *p* = 0.15 × 10^−3^) and colectomy-free rates reached 69% at 12 months [59]. Tacrolimus has a similar safety profile to cyclosporine with various side effects including infections, tremor and nephrotoxicity [59]. Current ECCO guidelines recognise tacrolimus as a possible alternative rescue therapy for ASUC [9], even though it is not widely used in clinical practice.

### Infliximab

Infliximab is a chimeric IgG1 monoclonal antibody specifically targeted against free and membrane-bound TNF-α which has proven efficacy in ASUC [60] and has become the most commonly used salvage therapy. In a randomised controlled trial by Järnerot et al., patients who were initially treated with IV betamethasone and received a single dose infliximab (5 mg/kg) as salvage therapy, had a significantly lower 3-month colectomy rate compared to placebo [61]. Long-term follow-up data showed that the benefit of rescue therapy with infliximab in ASUC remained after 3 years [62].

The standard induction regime for infliximab is 5 mg/kg at weeks 0, 2 and 6 [12] and for patients who have responded to infliximab, combination therapy with a thiopurine should always be considered, even in those who had previously failed thiopurine monotherapy [10]. There is evidence that combination therapy can result to increased blood levels of infliximab and decreased immunogenicity against infliximab [63].

Infliximab is contraindicated in congestive cardiac failure (New York Heart Association Class III/IV), demyelinating disease, active sepsis and latent tuberculosis and screening tests as described earlier should be performed prior to commencement of treatment [10]. Prophylactic therapy for *Pneumocystis jirovecii* should once again be considered for patients on triple immunosuppression. Infliximab may also have an impact on peri-operative complications and this needs to be taken into consideration, although data are mixed. Some studies did not show any significant increase in postoperative complications in patients with UC who had received infliximab pre-operatively [64], whereas others reported a worse postoperative morbidity since the introduction of biologics in therapy [65].

### Comparing infliximab and cyclosporine

Two head-to-head studies have compared efficacy of infliximab and cyclosporine for ASUC showing equivalence among the two medications. In the open-label CYSIF trial, there was no statistically significant difference between infliximab and cyclosporine in treatment failure, adverse events, mucosal healing rates, colectomy rates and colectomy-free survival at 1 and 5 years [66]. The CONSTRUCT trial was an open-label pragmatic randomised trial of infliximab and cyclosporine and again showed no significant difference in clinical effectiveness [67]. Moreover, a systematic review and meta-analysis of infliximab and cyclosporine randomised controlled trials showed no difference in response rates up to 1 year of therapy [68]. Cyclosporine and infliximab are equally effective in ASUC, but infliximab is simpler in use although more expensive [10]. The choice between rescue therapies should be individualised and several other factors, such as co-morbidities, contra-indications, previous exposure to medications, expertise and feasibility of laboratory testing, should be taken into consideration on decision [27].

### Sequential therapy in ASUC

Sequential therapy is defined as the use of a calcineurin inhibitor as rescue therapy in patients not responding to infliximab and vice versa. Sequential therapy is not recommended by current guidelines as it has been associated with serious side effects and infections because of cumulative immunosuppression [10, 40]. According to ECCO guidelines, only one attempt at rescue therapy should be considered before referral for colectomy and third-line medical therapy should only be considered in specialist referral centres and highly selected cases [9].

### Accelerated/intensified Infliximab dosing for ASUC

Two studies have demonstrated that higher infliximab serum concentrations are associated with greater efficacy and better outcomes [69, 70]. Moreover, the are several other factors that would support the need for dose optimisation of infliximab in the acute phase. These include the high TNF burden in ASUC, the proteolytic degradation of anti-TNF associated with increased drug clearance and faecal losses from increased gut permeability associated with severe inflammation [71]. According to BSG guidelines, patients who have not responded sufficiently 3–5 days after the first infliximab infusion should be treated with an accelerated induction regimen after a colorectal surgical review to exclude the need for emergency colectomy [10]. Some clinicians also use an initial 10 mg/kg dose as salvage therapy, however, optimal timing and dose are yet to be defined and further studies are needed for dose intensification to be implemented into clinical practice.

### Surgery for ASUC

The efficacy of rescue therapy in ASUC should be assessed daily [12] and although there have been no studies evaluating when rescue therapy should be considered unsuccessful, current guidelines recommend that patients with ASUC who have not responded within 7 days of rescue therapy require surgery [10, 12]. Colectomy should also be considered for patients who deteriorate prior to that time, or in case of complications, such as toxic megacolon, severe haemorrhage or perforation [10, 12]. Studies have shown that delayed surgery is associated with increased risk of complications [45] and that the only significant predictor of postoperative complications is a prolonged admission prior to surgery [72]. For this reason, it is imperative that decisions are made in a timely manner, in a multidisciplinary team setting, involving gastroenterologist, colorectal surgeon and stoma therapist if possible. Careful counselling is required so that surgical procedure, outcomes and possible complications are explained to the patient [12]. Colectomy should be considered as a treatment option and not an outcome that needs to be avoided at any cost [10]. The procedure of choice for patients with ASUC is total or subtotal colectomy with end ileostomy and preservation of rectum. The rectal stump may be managed by intraperitoneal closure or can be brought forward as a mucus fistula. The procedure can be performed open or laparoscopically based on local expertise. Subtotal colectomy is a safe procedure even in the setting of ASUC, it allows the patient to be relieved from disease burden, stabilize and recover. Moreover, it avoids pelvic dissection and intestinal anastomosis therefore minimising the potential for anastomotic leak. Definite histological confirmation can be obtained if needed and corticosteroids and immunosuppressant therapy can be weaned off prior to any further surgery. Restorative proctocolectomy with ileal pouch anal anastomosis (IPAA) is usually performed as a more definite procedure at a later stage [12, 26, 40].

### Emerging therapies

Two other anti-TNF agents, adalimumab and golimumab, and vedolizumab—a monoclonal antibody targeting the α_4_β_7_ integrin—have demonstrated efficacy in the treatment of moderate-to-severe UC [73–75]; however, there have been no specific studies for ASUC patients. Two recent studies have shown that vedolizumab can be effectively used as maintenance therapy in patients responding to calcineurin inhibitors [76, 77] and although further data are required, this can be kept into consideration in patients who had previously failed thiopurines and require cyclosporine as rescue therapy for ASUC. Tofacitinib is an oral Janus kinase (JAK) inhibitor which has proven effective in the treatment of moderate-to-severe UC. A recent post hoc analysis indicated a rapid onset of action with significant improvement on symptoms by day 3 of induction therapy with tofacitinib in UC [78]. Moreover, high-intensity tofacitinib was used successfully in 4 patients with ASUC [79]. Clearly, further randomised studies are required however, these data suggest that tofacitinib could potentially be used in the treatment of ASUC in the future. Finally, anakinra—IL-1 antagonist used for the treatment of patients with rheumatoid arthritis—is currently investigated as potential co-treatment with corticosteroids for ASUC [80].

## Conclusion

Acute severe ulcerative colitis is a medical emergency that warrants in-patient management. This is best served within a multidisciplinary team setting in specialised centres or with expert consultation. Intravenous corticosteroids remain the cornerstone in the management of ASUC and should be initiated promptly, along with general management measures and close monitoring of patients. Unfortunately, one-third of patients will fail to respond to steroids. Response to intravenous corticosteroid therapy needs to be assessed on the third day and rescue therapies, including cyclosporine and infliximab, should be offered to patients not responding. Choice of rescue therapy depends on experience, drug availability and factors associated with each individual patient, such as comorbidities, previous medications or contra-indications to therapy. Patients who have not responded within 7 days to rescue therapy must be considered for surgery. Surgery is a treatment option in ASUC and should not be delayed in cases of failure of medical therapy, because such delays increase surgical morbidity and mortality. Further research might help in defining predictors of response to salvage therapy and optimal dosing regimens, thus leading to a more personalised treatment for patients. Finally, further studies are required to investigate the potential benefit of other agents in the management of ASUC.
